# Assessing the utility of gene co-expression stability in combination with correlation in the analysis of protein-protein interaction networks

**DOI:** 10.1186/1471-2164-12-S3-S19

**Published:** 2011-11-30

**Authors:** Ashwini Patil, Kenta Nakai, Kengo Kinoshita

**Affiliations:** 1 Human Genome Center, Institute of Medical Science, The University of Tokyo, 4-6-1 Shirokane-dai, Minato-ku, Tokyo 108-8639, Japan; 2 Graduate School of Information Sciences, Tohoku University, 6-3-09, Aramaki-aza-aoba, Aoba-ku, Miyagi, 982-0036, Japan; 3 Bioinformatics Research and Development, Japan Science and Technology Corporation, 4-1-8 Honcho, Kawaguchi, Saitama 332-0012, Japan

## Abstract

**Background:**

Gene co-expression, in the form of a correlation coefficient, has been valuable in the analysis, classification and prediction of protein-protein interactions. However, it is susceptible to bias from a few samples having a large effect on the correlation coefficient. Gene co-expression stability is a means of quantifying this bias, with high stability indicating robust, unbiased co-expression correlation coefficients. We assess the utility of gene co-expression stability as an additional measure to support the co-expression correlation in the analysis of protein-protein interaction networks.

**Results:**

We studied the patterns of co-expression correlation and stability in interacting proteins with respect to their interaction promiscuity, levels of intrinsic disorder, and essentiality or disease-relatedness. Co-expression stability, along with co-expression correlation, acts as a better classifier of hub proteins in interaction networks, than co-expression correlation alone, enabling the identification of a class of hubs that are functionally distinct from the widely accepted transient (date) and obligate (party) hubs. Proteins with high levels of intrinsic disorder have low co-expression correlation and high stability with their interaction partners suggesting their involvement in transient interactions, except for a small group that have high co-expression correlation and are typically subunits of stable complexes. Similar behavior was seen for disease-related and essential genes. Interacting proteins that are both disordered have higher co-expression stability than ordered protein pairs. Using co-expression correlation and stability, we found that transient interactions are more likely to occur between an ordered and a disordered protein while obligate interactions primarily occur between proteins that are either both ordered, or disordered.

**Conclusions:**

We observe that co-expression stability shows distinct patterns in structurally and functionally different groups of proteins and interactions. We conclude that it is a useful and important measure to be used in concert with gene co-expression correlation for further insights into the characteristics of proteins in the context of their interaction network.

## Background

mRNA expression information is often used in combination with protein-protein interaction networks in order to provide a better perspective on proteins and their inter-relationships in the cell. mRNA co-expression of genes across various conditions is quantified in the form of a correlation coefficient of their expression levels across multiple samples. Co-expression correlation has been used in the prediction of protein-protein interactions [1], though with limited success [2]. Other studies have used the combination of protein-protein interaction information and gene co-expression correlation to identify functional modules of proteins that are active in a particular disease state [3,4], the rate of evolution of proteins [5], and the levels of disorder in co-regulated proteins [6]. It has also been used as the primary means of classifying hub proteins in protein-protein interaction (PPI) networks into date hubs and party hubs [7], or inter-modular and intra-modular hubs [8], independently and in combination with gene expression stability [9,10]. In spite of being widely studied, this classification has not been replicated [11,12] and gene co-expression correlation as a sole means of classifying hubs has been shown to be unreliable [13], stressing the need for the use of additional information.

Undoubtedly, gene co-expression correlation is an important characteristic when used in the context of protein-protein interaction networks. However, it is often biased due to disproportionately large contributions of a few samples [14]. For instance, genes that are expressed in the same tissue often show a misleadingly high correlation coefficient in spite of the lack of a functional relationship. Gene co-expression stability quantifies the bias in the correlation coefficient by indicating the change in co-expression of a pair of genes on the removal of samples contributing most to the correlation coefficient. It has been shown that genes with high stability are functionally related in spite of low correlation coefficients. On the other hand, those with low stability have fragile co-expression which implies limited, or no functional relation [14]. Thus, the co-expression stability may be viewed as a reliability measure of the co-expression correlation coefficient. The combination of correlation and stability represents the co-expression of genes across multiple samples along with the amount of bias there-in.

In this study, we investigate the usefulness of the gene co-expression stability in concert with co-expression correlation in the analysis of various characteristics of gene pairs in the context of the human protein-protein interaction network. Specifically, we study the relationship of gene co-expression correlation and stability with the interaction promiscuity of proteins, their levels of intrinsic disorder and their tendency towards essentiality and disease-relatedness. We demonstrate that the gene co-expression stability is a useful means of distinguishing different kinds of proteins in the protein-protein interaction network and can be used with the co-expression correlation coefficient for more effective analysis.

## Results

In order to evaluate the utility of gene co-expression stability in combination with co-expression correlation coefficient, we used a high confidence human protein-protein interaction network from the database, HitPredict [15]. Gene co-expression correlation coefficients were calculated for interacting protein pairs over 18800 samples from the Gene Expression Omnibus [16] and normalized using the MAS5 algorithm. Stability was calculated as shown in Kinoshita and Obayashi [14] and briefly described in the Methods (Equation 1). Genes pairs with co-expression correlation coefficient less than 0.2 were ignored since the stability measure was not found to be sufficiently informative below this threshold. This gave a dataset of 8182 interactions among 3715 proteins. We looked at various properties of the proteins and the interactions in relation to their gene co-expression correlation and stability.

### Co-expression correlation and stability in the protein-protein interaction network

**Figure 1:**
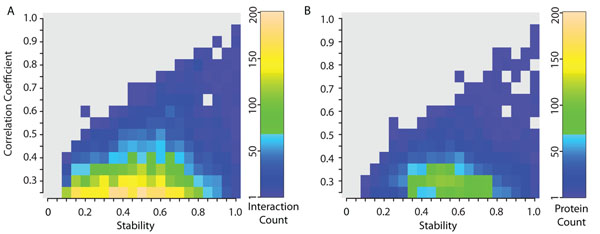
**Distribution of interactions and proteins over co-expression correlation and stability.** Number of a) interactions and b) proteins in the human protein-protein interaction network within ranges of co-expression correlation coefficient and stability as indicated in the heat map. The values for interactions are those of the gene pairs of interacting proteins. The values for proteins are averaged across all the interactions they participate in. The absence of proteins or interactions (0 value) is indicated in gray in the heat map to allow for better visualization of outliers. The correlation between co-expression correlation and stability is low showing their independence as sources of information.

We studied the relationship between co-expression correlation and stability in pairs of interacting proteins (Figure 1a). Correlation coefficient and stability are, in general, not highly correlated (Spearman’s rank correlation = 0.197, p < 0.0001) and thus provide independent sources of information about interacting proteins. Most interacting proteins pairs have a low co-expression correlation coefficient, making it a poor predictor of physical interactions among proteins, as has been previously observed [2]. It is notable that most interacting proteins with large co-expression correlation coefficients (0.5 or greater) also have large stability values, with almost none having stability values below 0.5. These primarily represent interactions between members of stable protein complexes like the subunits of the proteasome degradation complex, or subunits of the replication helicase MCM complex. We study these outliers with special interest in the later analyses. Most interactions with co-expression correlation less than 0.5 show varying levels of stability. Low stability values in these interactions are indicative of high levels of bias and fragile co-expression correlation coefficients, which must be used with caution.

The average co-expression correlation coefficient and stability for each protein were calculated as shown in Equations 2 and 3, respectively (See Methods). Proteins show a distribution that is similar to interactions in the correlation coefficient and stability landscape (Figure 1b). Co-expression correlation and stability show no correlation (Spearman's rank correlation=0.013, p=0.22). There are, however, a few outliers with larger values of correlation and stability. Average co-expression correlation coefficients for proteins with low stability must be used with caution due to the large amount of bias.

### Hubs and hub types in the protein-protein interaction network

**Figure 2:**
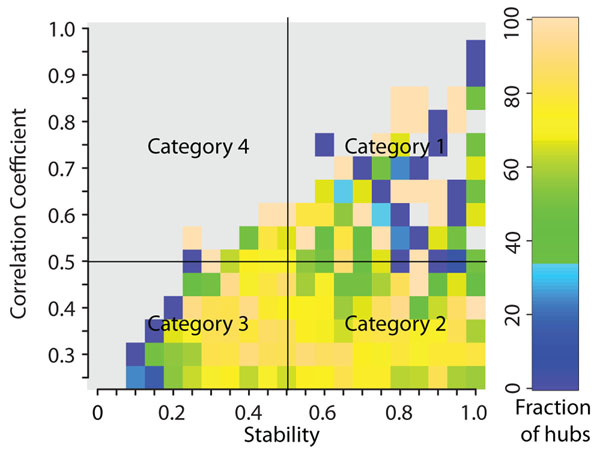
**Prevalence and classification of hub proteins using co-expression correlation and stability.** Frequency of hubs in proteins with varying levels of co-expression correlation and stability with their interaction partners. Gray regions indicate the absence of proteins for that window of correlation and stability values. Hub proteins are divided into 4 categories as shown. Category 1 – correlation > 0.5, stability > 0.5; Category 2 – correlation <= 0.5, stability > 0.5; Category 3 – correlation <= 0.5, stability <= 0.5; Category 4 – correlation > 0.5, stability <= 0.5. Refer Figure S1c in Additional File 1 for standard error values.

Figure 2 shows the prevalence of hubs (proteins with 5 or more interactions) as a fraction of proteins within a specified range of average co-expression correlation and stability. Most hubs lie in areas of low co-expression correlation with their interaction partners and relatively high stability (Figure S1a,b in Additional File 1). These hubs appear to participate primarily in transient interactions. A small number of hubs with high average co-expression correlation and high stability are constitutively expressed with their interaction partners. The phenomenon of fragile co-expression is very rare in the interaction network as evidenced by the small number of hubs with high co-expression correlation and low stability.

The classification of hubs is a widley studied problem. Hubs have primarily been classified into transient (date, inter-modular) and obligate (party, intra-modular) hubs using co-expression correlation alone [7,8]. However, these results are disputed [11-13]. They have also been classified using protein structure [17] and gene expression stability [9]. In spite of the various methods used, there is no consensus in the classification of hubs. We tested whether the previous classification of hubs is robust and if the stability measure can be used with the co-expression correlation coefficient to classify hubs into functionally independent groups. To perform this analysis, we divided hubs into 4 categories based on their correlation and stability values, and studied the differences in their network characteristics and functional annotations. Hubs were identified as proteins with at least 5 interactions within a particular category (Refer Table 1). We ignored hubs in category 4 (correlation > 0.5 and stability < = 0.5) in our analysis since it contained only 4 hubs. We looked at the network characteristics of the hubs in the 3 categories in the form of the clustering coefficient and the betweenness centrality. The clustering coefficient indicates the level of connectivity between the partners of a protein, with high values corresponding to intra-modular proteins [18]. On the other hand betweenness centrality is a measure of the number of shortest paths that go through the protein with higher values indicating inter-modular proteins [19]. Average values of clustering coefficients and betweenness centrality were calculated using Equations 4 and 5 (See Methods).

**Table 1:** Network characteristics of hub proteins in 3 categories.

Type	Average clustering coefficient	Average betweenness centrality (10^-4^)
Category 1 (41 hubs)	0.231 ±0.017*	5.56 ±0.53
Category 2 (264 hubs)	0.099 ±0.016	36.32 ±2.41
Category 3 (315 hubs)	0.154 ±0.004	10.91 ±2.79

Average clustering coefficient and betweenness centrality for 3 categories of hubs based on co-expression correlation and stability. Category 1 – correlation > 0.5, stability > 0.5; Category 2 – correlation <= 0.5, stability > 0.5; Category 3 – correlation <= 0.5, stability <= 0.5. Differences in the distributions of Hub categories 1, 2 and 2, 3 are statistically significant at p << 0.001.

* 95% confidence intervals for average clustering coefficient and betweenness centrality for hubs in each category.

Hubs in Category 1 have a high clustering coefficient and low betweenness centrality (Table 1, See also Figure S2 in Additional File 1). These hubs have a high co-expression correlation and a high stability with their interaction partners. Taken together, this implies that hubs in Category 1 correspond to obligate hubs or intra-modular hubs that are parts of complexes and constitutively expressed with their interaction partners. A Gene Ontology (GO) term enrichment analysis confirms this result with significantly enriched terms like DNA replication initiation, DNA replication checkpoint, proteasome core complex, MCM complex, etc (Tables S2-S4 in Additional File 1). Examples of category 1 hubs include proteasome complex subunits and ORC subunits among others.

On the other hand, Category 2 hubs, which have a low co-expression correlation and high stability, have low a clustering coefficient and high betweenness centrality indicating their inter-modular nature. The low co-expression correlation of these hubs denotes the ability to participate in transient interactions. The high stability values show low levels of bias in the correlation coefficients. These hubs are significantly enriched for GO terms like Ras protein signal transduction, ATP binding and transcription factor complex among others (Tables S2-S4 in Additional file 1), signifying roles in signal transduction and transcription regulation. BRCA2, p53 and NF kappa B are some of the hubs in category 2. Categories 1 and 2 correspond to the party and date hubs respectively, as proposed by Han et al. [7]. This distinction is further supported by the fact that hubs in both these categories show high co-expression stability indicating that their co-expression correlation coefficients are not fragile.

Hubs in category 3 have low co-expression correlation and low stability with their interaction partners. The low co-expression correlation and stability indicates high variation in co-expression and fragile correlation coefficients. These hubs have network characteristics that are intermediate to those of category 1 and 2 hubs, with low clustering coefficient but also low betweenness centrality. This indicates that the hubs in category 3 are neither inter-modular, nor intra-modular, but belong to an entirely different class. GO term analysis confirms this result by showing significantly enriched terms like nuclear mRNA splicing via spliceosome, mRNA transport and RNA binding, spliceosome (Tables S2-S4 in Additional file 1). This class includes several small nuclear ribonucleoproteins. In spite of their inherent functional differences, the hubs in categories 2 and 3 are often combined into a single class of transient (date, inter-modular) hubs in classification systems using average co-expression correlation coefficient alone. The use of stability helps separate these hubs further into two functionally distinct groups.

This result demonstrates the ability of the stability measure as an information source that is independent of the co-expression correlation coefficient. More importantly, this analysis shows that the currently accepted classification of hubs into just two types -transient and obligate - using co-expression correlation coefficient alone, is insufficient to separate the many functionally distinct groups that exist in the PPI network. Using different measures along with the co-expression correlation coefficient will improve the identification of these groups.

### Intrinsic disorder in interacting proteins

**Figure 3:**
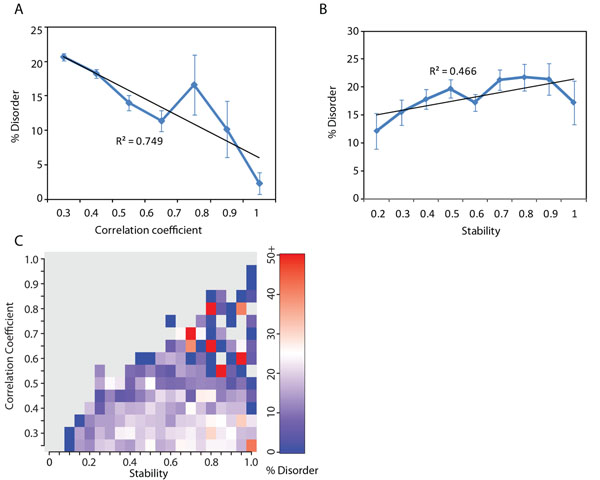
**Relationship between average intrinsic disorder in proteins and their co-expression correlation and stability.** Average percentage of intrinsic disorder in proteins as compared to the average co-expression a) correlation, and b) stability calculated over all interactions. c) Distribution of the fraction of disordered residues for proteins over varying levels of average co-expression correlation and stability. Gray regions indicate the absence of proteins for that window of correlation and stability values. Refer Figure S3 a in Additional File 1 for standard error values. Proteins that have low co-expression correlation and high co-expression stability with their interaction partners contain the highest levels of disorder.

Intrinsic disorder has been extensively studied in protein-protein interaction networks [20-23]. Its relationship with gene expression was studied by Edwards et al. who found that high levels of disorder are associated with low levels of gene expression, expect for a few highly disordered proteins [6]. Here, we investigated if co-expression stability information provides new insights in the co-expression patterns of disordered proteins. We studied the average levels of intrinsic disorder in proteins for various values of co-expression correlation and stability (Figure 3). Figure 3a shows an inverse relationship between intrinsic disorder and co-expression correlation in proteins (Spearman’s rank correlation=-0.109, p < 0.0001). Proteins with high levels of intrinsic disorder have low average co-expression correlation with their interaction partners (Figure S3b in Additional File 1). These proteins also show, on average, higher stability than ordered proteins (Figure 3b, p < 0.0001. Refer Figure S3b in Additional File 1). Thus, these proteins participate in transient interactions with robust co-expression correlation coefficients. They include the hubs in Category 2. The amounts of proteins with high levels of intrinsic disorder are known to be tightly regulated in the cell through the regulation of their transcript levels [24,25], which suggests their participation in transient interactions. The importance of the role played by intrinsic disorder in transient protein-protein interactions has been extensively studied [26]. The heat map in Figure 3c provides further insights. It shows that the levels of intrinsic disorder are also high in a few proteins having high co-expression correlation and stability with their interaction partners. These proteins participate in obligate interactions as members of complexes and include hubs in category 1. Though the number of such proteins is small, their characteristics appear to be very distinct. These results are also in agreement with an earlier study by Higurashi et al. who found high levels of intrinsic disorder in stable, complex-forming hubs [17]. Thus, our results support the previously suggested hypothesis that proteins with high levels of disorder are either tightly regulated and participate in transient interactions, or are constitutively expressed and exist as subunits of stable complexes [25].

Finally, when combined with the previously described categories in hubs, this result shows that not all hubs have high levels of intrinsic disorder. Specifically, hubs in categories 1 and 2 show high levels of intrinsic disorder. On the other hand, hubs in category 3, which have fragile co-expression correlation, show low levels of disorder. It is possible that the fragile patterns of co-expression are not conducive to the presence of large disordered regions in these proteins. Thus, with the help of co-expression stability and correlation information, we can conclude that the amount of intrinsic disorder affects the expression patterns of hubs, and proteins, in general.

### Interactions between ordered and disordered proteins

Given the differences in the levels of co-expression patterns of proteins with high levels of intrinsic disorder, we examined these patterns for interactions between proteins with high or low levels of disorder. We specifically looked at distributions of co-expression correlation and stability for interactions where both interacting proteins have high levels of intrinsic disorder (intrinsic disorder >= 30%), one protein has high levels of intrinsic disorder, and both proteins are ordered (intrinsic disorder < 30%).

**Figure 4:**
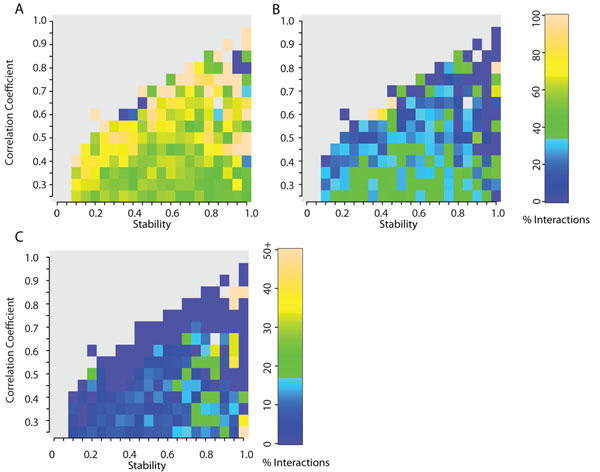
**Co-expression correlation and stability landscape for interactions between ordered and disordered proteins.** Frequency of interactions in the context of co-expression correlation and stability of the interacting proteins, where a) both proteins are ordered and contain less than 30% disordered residues, b) one protein is disordered with more than 30% disordered residues, while the other is ordered, and c) both proteins are disordered and contain more than 30% disordered residues. Lighter colors indicate greater prevalence and gray indicates an absence of interactions within that range of co-expression correlation and stability. Refer Figure S4 in Additional File 1 for standard error values for all heat maps. The 3 types of interactions show distinct patterns highlighting the differences in the expression patterns of the genes encoding these proteins.

Figure 4 shows the distinct patterns of co-expression correlation and stability made by each of the three types of interactions. The co-expression patterns of two largely disordered interacting proteins and two largely ordered ones shows the greatest difference. Disordered protein pairs show lower co-expression correlation and higher stability as compared to ordered protein pairs (Figure S5 in Additional File 1, p<0.001). An example is the interaction between two largely disordered proteins, the nuclear receptor coactivator NCOA6, and the histone acetyl transferase CREB-binding protein (CREBBP), which is thought to result in transcriptional activation. The low co-expression and high stability suggest transient interactions which in turn may be the effect of tighter regulation of disordered proteins. The heat map in Figure 4C also shows a small population of interacting disordered proteins with high co-expression correlation and stability indicative of obligate interactions like that between the Jun and Fos proteins, or Jun and AP1 both of which function in transcription regulation. Interactions between ordered and disordered proteins also show low co-expression correlation but with low average stability. These properties are primarily associated with transient interactions with fragile co-expression correlation coefficients.

These results show that interacting protein pairs with varying levels of intrinsic disorder show distinct patterns of not only co-expression correlation, but also stability, being either constitutively or transiently expressed with their partner proteins.

### Essential and disease genes

**Figure 5:**
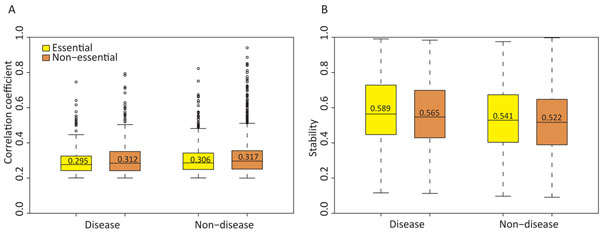
**Correlation coefficient and stability for disease and essential genes.** Distribution of a) co-expression correlation and b) stability for disease, essential, non-disease and non-essential genes. The average values are specified. The differences in correlation and stability distributions of “Disease essential” and “Non-disease non-essential” are statistically significant at p = 2.319e-5 and p=6.39e-9, respectively. These differences are also significant for “Essential” compared to “Non-essential”, and “Disease” compared to “Non-disease” at p <0.001. Statistical significance was calculated by the Wilcoxon rank sum test.

The co-expression patterns of disease and essential genes in the human PPI network have been extensively studied [3,4]. We identified disease and essential genes in the PPI network as in Goh et al. [3] (See Methods). Figure 5 (Figure S6 in Additional File 1) shows the average co-expression correlation and stability of disease and essential genes with their interaction partners in the PPI network. Disease genes have a lower average co-expression correlation and a higher average stability than non-disease genes (p < 0.0001). Essential and non-essential genes also show a similar pattern (p < 0.0001). Essential disease genes show the lowest co-expression correlation and highest stability, while non-essential non-disease genes show the lowest stability and highest co-expression correlation (p < 0.0001). The pattern of low co-expression correlation and high stability in disease and essential genes is indicative of transient interactions with correlation coefficients that are not biased or fragile. Thus, different types of genes not only have distinct patterns of co-expression but also of stability. Finally, non-essential disease genes have high co-expression correlation and stability with their interaction partners suggesting their participation in obligate interactions.

**Figure 6:**
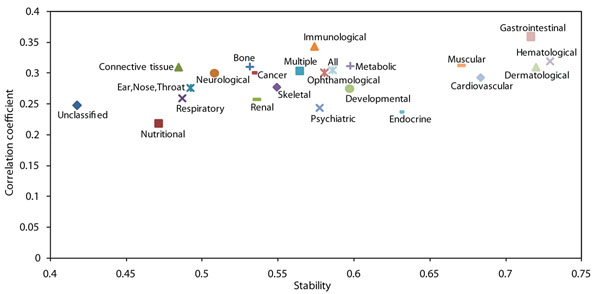
**Average co-expression correlation and stability over disease classes.** Average co-expression correlation and stability of genes implicated in different diseases with their interaction partners. The co-expression stability shows relatively more variation than the co-expression correlation. See Figure S8 in Additional File 1 for standard error values.

For a more detailed analysis of the correlation and stability patterns of genes in various types of diseases, we divided the disease genes into distinct classes as given by Goh. et al. [3]. We found that though the average correlation coefficient of these genes with their interaction partners is similar (average 0.3), the co-expression stability shows relatively greater variation (Figure 6 and Figure S7 in Additional File 1). For example, the genes implicated in neurological diseases have a low average co-expression stability as compared to those implicated in hematological diseases (Figure S8 in Additional File 1) demonstrating that the genes responsible for neurological diseases show fragile co-expression patterns with their interaction partners, as compared to those implicated in hematological diseases.

Thus, co-expression stability provides additional information about genes and their functions when used with gene co-expression correlation.

## Discussion

Gene co-expression stability has been used to identify the functional relationship between pairs of co-expressed genes in *Arabidopsis thaliana*[14]. However, functional relation is a foregone conclusion in the context of two interacting proteins. Hence, we tried to assess the utility of co-expression stability of interacting proteins in order to elucidate the relationships between proteins and the nature of their interactions. This is an important aspect of the study of PPI networks, since the current static data of protein interactions does not necessarily reflect the spatial and temporal relationships between the interacting proteins under physiological conditions. Our primary goal throughout this study has been to look for specific patterns of stability in distinct groups of proteins and interactions, which are separate from their patterns of co-expression correlation. We were able to find such differences in several groups of proteins and interactions, allowing us to conclude that stability is an informative measure, which when used in combination with co-expression correlation, provides information that is otherwise inaccessible.

A case in point is the identification of a class of hubs having characteristics that are distinct from the currently accepted transient and obligate hubs. Not only does this result highlight the usefulness of the stability measure, but it also shows the insufficiency of using the co-expression correlation alone as a means of classifying hubs. Different measures like stability are needed to broaden this classification. Gene expression stability has been proposed as one such measure [9], as is simple GO term based classification [13].

The distinct patterns of co-expression correlation and stability for proteins with different levels of intrinsic disorder, and different disease annotations, further confirm the utility of using the combination of correlation and stability. This also leads to new insights about the proteins and their properties. For instance, we find that proteins with low co-expression stability have low levels of intrinsic disorder. In another example it is observed that non-essential disease genes primarily participate in obligate interactions as indicated by their high correlation and stability. Such inter-relationships are easily elucidated through the combined usage of correlation coefficients and stability.

Other measures that have been similarly used in combination with the co-expression correlation include the gene expression variability and the rank of co-expression correlation. The gene expression variability, in the form of standard deviation, has been successfully used to classify hubs [9] and identify selective gene expression patterns [27]. Rank of co-expression correlation between genes has also been used to address the issue of bias in co-expression correlation. The absolute values of correlation often change with the samples used for calculation making it difficult to introduce a single threshold value to determine significantly correlated gene pairs. The rank of correlation provides a solution for this problematic bias. It works as a better indicator of functionally related genes than the correlation coefficient [28]. Since rank of correlation is a general approach, multidimensional correlation – that have been used to calculate the stability - can be converted into multidimensional rank by considering the rank of correlation in each dimension. We have not checked the efficiency of the multidimensional rank, but it will be interesting to compare the results obtained using the stability measure with rank measures as well.

It is also conceivable to use the stability measure as a parameter in prediction studies along with the co-expression correlation, either in the prediction of different classes of proteins, like disordered or ordered, or those that are active in different diseases or functional modules. The gene co-expression stability is an extensible and easily accessible measure. Values for gene co-expression stability can be obtained for several species, including human, from COXPRESdb [29], and for *Arabidopsis thaliana* from ATTEDII [30]. In this study, we have limited ourselves to assessing the utility of this measure. However, each of the findings needs to be explored independently in greater detail.

## Conclusions

We assessed the utility of the gene co-expression stability as a measure for further understanding the properties of proteins and their inter-relationships within the human protein-protein interaction network, in combination with gene co-expression correlation. We demonstrate that different types of proteins and interactions not only show distinct patterns of co-expression correlation but also of co-expression stability. We show the inadequacy of co-expression correlation as a means of classifying hubs and find that stability improves its performance. Specifically, we identify transient and obligate hubs along with a previously unknown type that is functionally distinct. Other patterns that we elucidated include low co-expression correlation and high stability of protein with high levels of intrinsic disorder. This combination of parameters also gives distinct co-expression patterns for pairs of interacting proteins that are highly ordered or disordered. We also show that disease and essential genes have very high co-expression stability and thus stable co-expression patterns with their interaction partners, independent of their co-expression correlation. Finally, we show that genes in different classes of diseases have distinct co-expression stability providing a possible means of distinguishing them based on co-expression and interaction patterns. Thus, we show that gene co-expression stability is a useful measure to be used in concert with co-expression correlation and provides additional information leading to a better understanding of proteins in PPI networks. Future prospects include studying each of the results obtained here in greater detail, comparing our results with other measures of gene co-expression stability, as well as implementing a predictor using this combination in the prediction of membership of proteins to distinct classes.

## Methods

High confidence human protein-protein interactions were taken from the HitPredict [15] database. Hubs within the entire network were denoted as proteins with 5 or more interactions. This definition of hubs has previously been shown to be robust [31]. Hubs in each category based on gene expression correlation and stability, were denoted as proteins having 5 or more interacting partners with whom they show specific levels of co-expression correlation and stability as required by the category cutoffs.

Gene expression correlation coefficients and gene expression stability values were calculated as described in Kinoshita and Obayashi [14]. Gene expression correlation coefficients were calculated for interacting protein pairs over 18800 human samples obtained from the Gene Expression Omnibus [16]. These were normalized using the MAS5 algorithm in R. Principal Component Analysis (PCA) was performed in sample space and the resulting PCs were obtained. The correlation coefficient (cor0) was calculated in PC space, as the Pearson’s Correlation Coefficient (PCC), using the top 3894 PCs which corresponded to 80% of the variation in gene expression. This cutoff was chosen based on data from the previous study which showed that only 23.8% of the PCs represent 80% of the variation in gene expression followed by a rapid decline in the contribution of the PCs [14]. Subsequently, 10 correlation coefficients (cor1, cor2, cor3 ..., cor 10) were calculated on the removal of the 1^st^, 2^nd^, 3^rd^, ... 10^th^ PC. The top 10 PCs were chosen since they approximately correspond to the number of “informative experiments” as previously suggested [32]. These correlation coefficients were then used to calculate the co-expression stability using the formula obtained from [14], as shown below in equation 1.(1)

where *cor_i_* is the correlation without the first *i* PCs, *cor_max_* is the maximum value from *cor_0_* to *cor_10_*, *i* = 0*..N*, and *N* = 10.

Pairs of genes with *cor_0_* less than 0.2 were ignored since the stability measure for these was not found to be sufficiently informative. Using protein pairs in the interaction network with co-expression correlation and stability values, resulted in 8182 interactions among 3715 proteins.

Average co-expression correlation coefficient for a protein is calculated as follows:(2)

where *n* = number of interactions of the protein,

*r_ai_* = co-expression correlation coefficient in PC space (cor0 in equation (1)) for genes of protein *a* and it’s *i^th^* interaction partner

Average stability for a protein was similarly calculated as:(3)

where *n* = number of interactions of the protein,

*S_ai_* = co-expression stability for genes of protein *a* and it’s *i^th^* interaction partner as calculated by equation (1)

Clustering coefficient and betweenness centrality for each protein in the PPI network were calculated using the Netanalyzer plugin [33] in Cytoscape [34].

Average clustering coefficient for hubs in a category *i*, *i* = 1..3, was calculated as:(4)

where *N* = number of hubs in category *i*,

*CC_j_* = clustering coefficient of hub *j* in category *i*

Similarly average betweenness centrality for hubs in a category *i*, *i* = 1..3, was calculated as:(5)

where *N* = number of hubs in category *i*,

*BC_j_* = clustering coefficient of hub *j* in category *i*

Significantly enriched Gene Ontology (GO) terms [35] for Biological Process, Molecular Function and Cellular Component in each category of hubs were determined separately using the hypergeometric distribution at a significance level of p < 0.01.

Intrinsic disorder was predicted in all proteins using the program metaPrDOS [36] at a false positive rate of 5%. Regions with 30 consecutive residues predicted as disordered were considered as disordered regions. Interaction types were assigned based on the intrinsic disorder content in the interacting proteins. Table S5 in Additional File 1 gives the number of interactions in each type.

Disease and essential genes were obtained as in Goh et al. [3] Disease annotations for proteins in the PPI network were obtained from the Online Mendelian Inheritance in Man (OMIM) [37]. Essential genes were identified as orthologs of mouse genes whose disruption was lethal in the embryonic or postnatal stages, as obtained from Mouse Genome Informatics (MGI) [38]. Disease classes, as given by Goh et al. [3], were assigned to disease genes. The number of disease and essential genes found in the dataset are shown in Table S6 in Additional File 1.

## List of abbreviations

PPI: Protein-protein interaction; GO: Gene Ontology; PCA: Principal component analysis; PC: Principal component; PCC: Pearson’s correlation coefficient.

## Authors’ contributions

AP and KK conceived of the study, prepared raw data, analyzed results and drafted the manuscript. KN analyzed results and provided computational resources. All authors read and approved the final manuscript.

## Competing interests

The authors declare that they have no competing interests.

## Supplementary Material

Additional File 1 **Supplementary materials** Supplementary figures and tables. Click here for file
