# Supplementary material for: Assessing the utility of gene co-expression stability in combination with correlation in the analysis of protein-protein interaction networks
Source: BMC Genomics. 2011 Nov 30;12(Suppl 3):S19. doi: 10.1186/1471-2164-12-S3-S19 (PMC3333178; doi:10.1186/1471-2164-12-S3-S19)
Supplement: Additional File 1 — Supplementary materials Supplementary figures and tables. [file 1471-2164-12-S3-S19-S1.pdf]

Supplementary Materials

Supplementary Figures

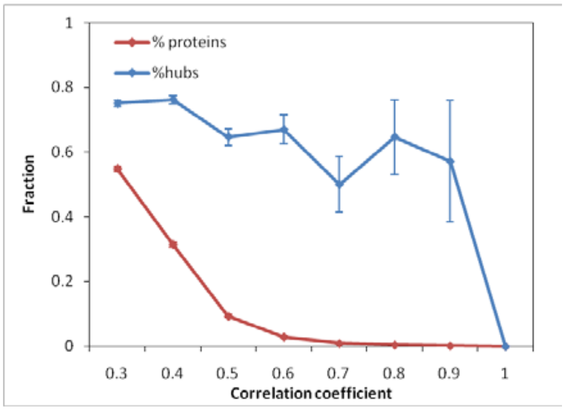

a)

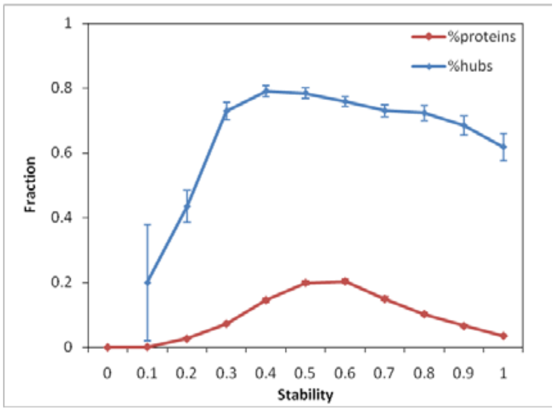

b)

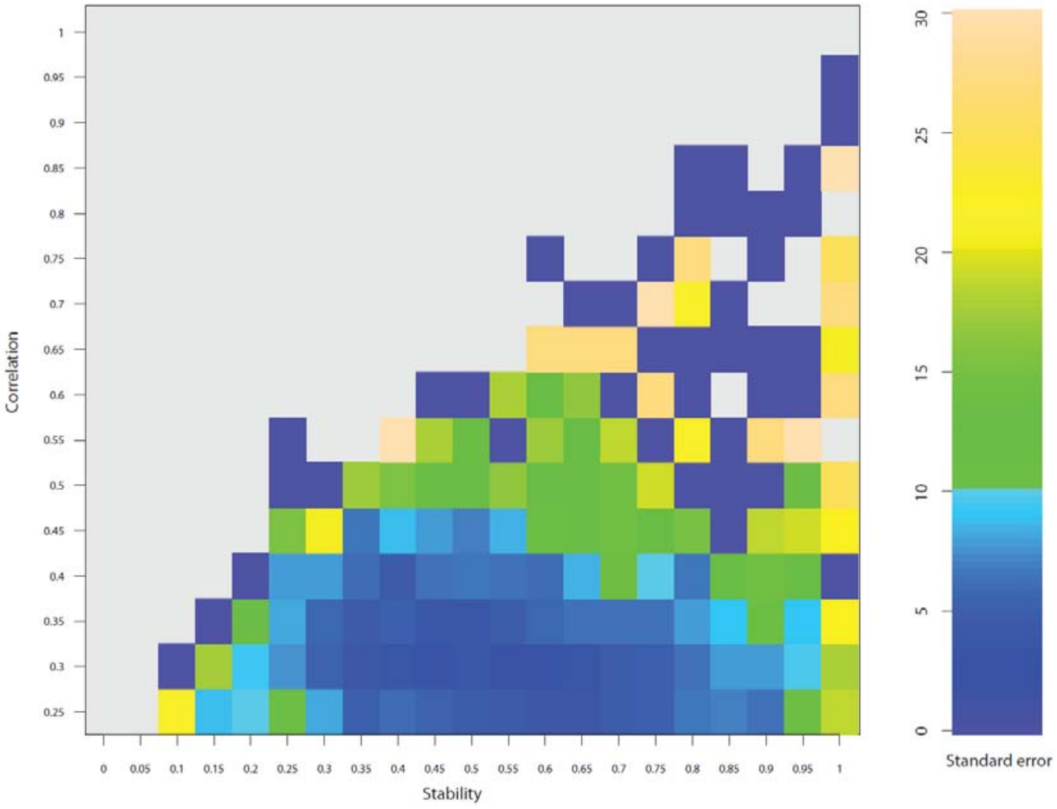

c)

Figure S1. Frequency of proteins and the prevalence of hubs in them, at varying levels of the average co-expression a) correlation and b) stability of proteins with their interaction partners. c) Standard error for frequency of hubs in heatmap shown in Figure 2. Gray regions indicate undefined values.

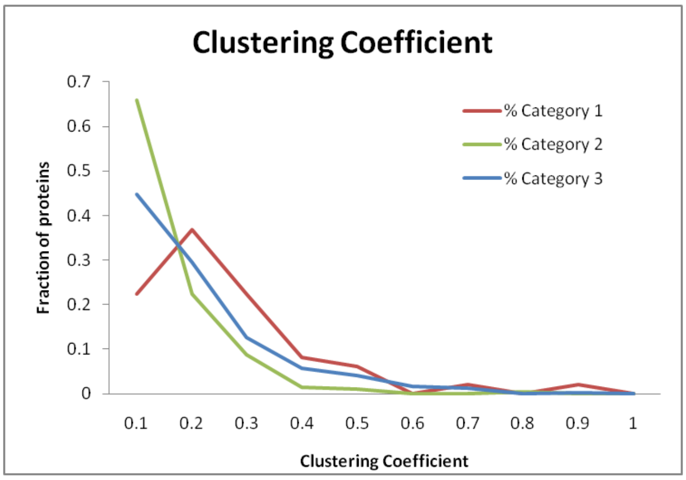

a)

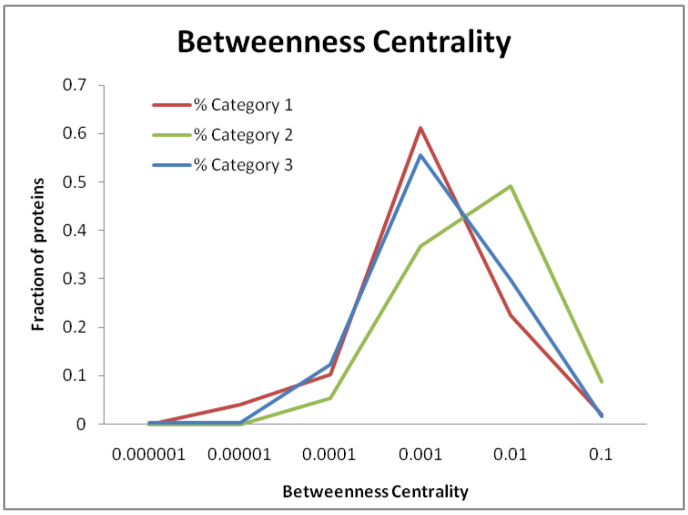

b)

Figure S2. Distribution of a) Clustering coefficient (CC) and b) Betweenness centrality (BC) for three categories of hubs as defined by their average coexpression correlation and stability with their interaction partners. Category 1 – correlation > 0.5, stability > 0.5; Category 2 – correlation <= 0.5, stability > 0.5; Category 3 – correlation <= 0.5, stability <= 0.5. All differences in the distributions of BC and CC are statistically significant at p << 0.001.

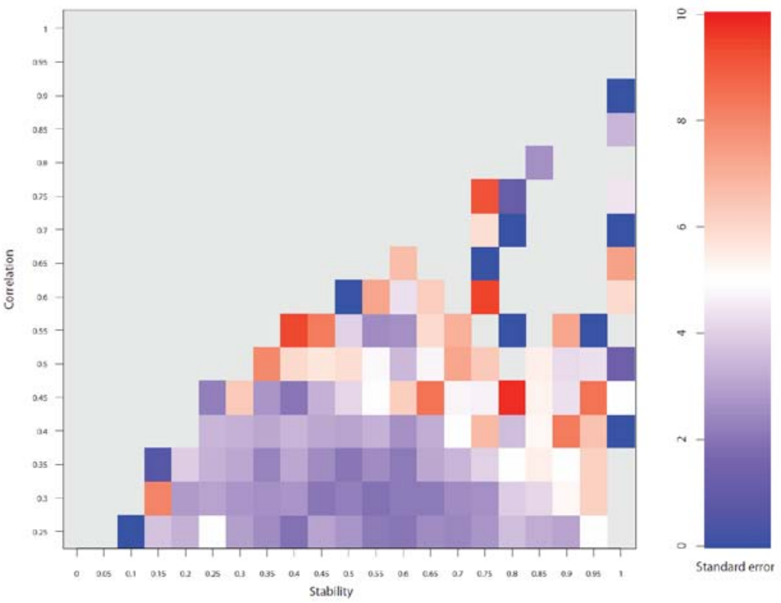

a)

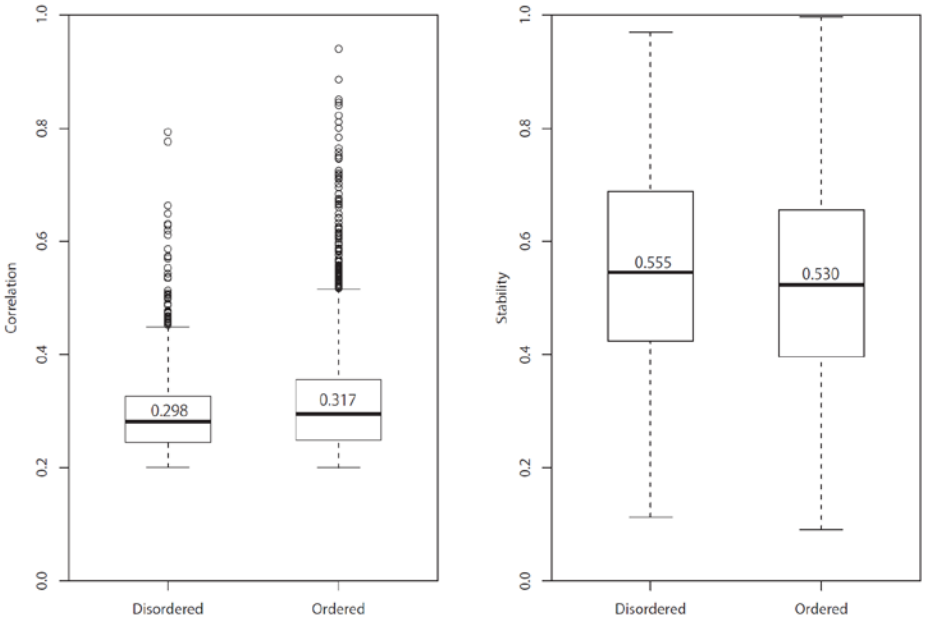

b)

Figure S3. a) Standard error in average % disorder for proteins in a given window of correlation and stability as shown in Figure 3c. Gray regions indicate undefined values. b) Correlation coefficient and stability of disordered proteins (disorder >= 30%) and ordered proteins (disorder < 30%) with average values specified in the boxes. Differences between average correlation and stability in disordered and ordered proteins are statistically significant at $p = 1.745e^{-6}$ and $p = 6.417e^{-4}$, respectively (Wilcoxon rank sum test).

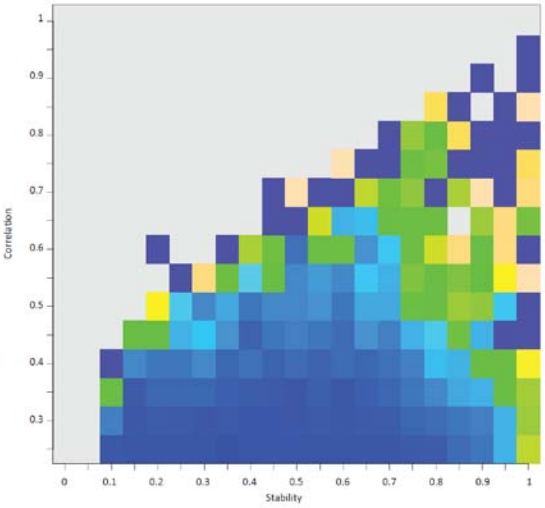

a)

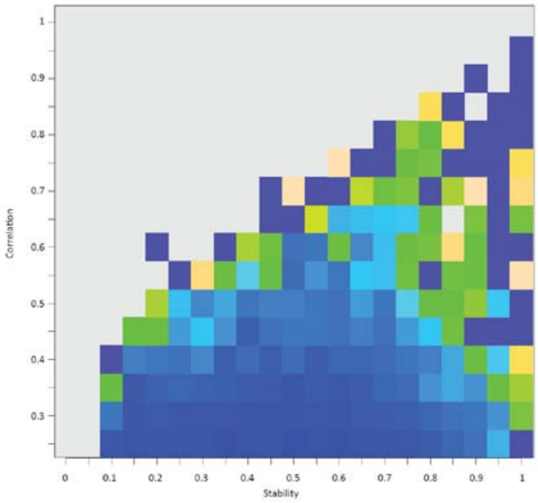

b)

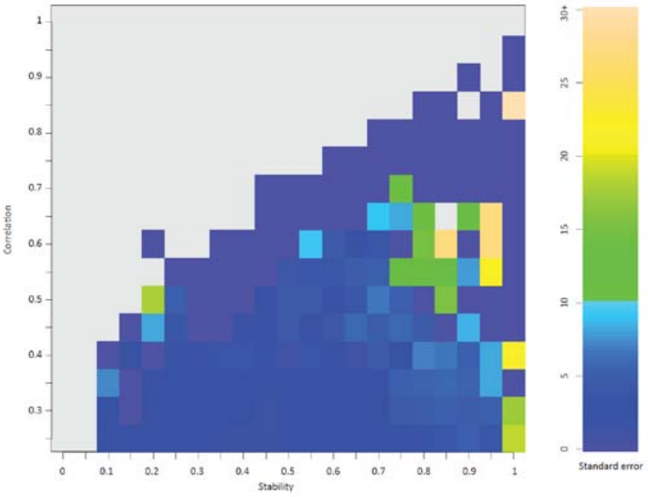

c)

Figure S4. Standard error values for the fraction of interactions in a given window of correlation and stability as shown in Figure 4 for types a) O-O: both interacting proteins have less than 30% intrinsic disorder, b) D-O: one of the interacting proteins has >= 30% intrinsic disorder and c) D-D: both interacting proteins have >=30% intrinsic disorder. Gray regions indicate undefined values.

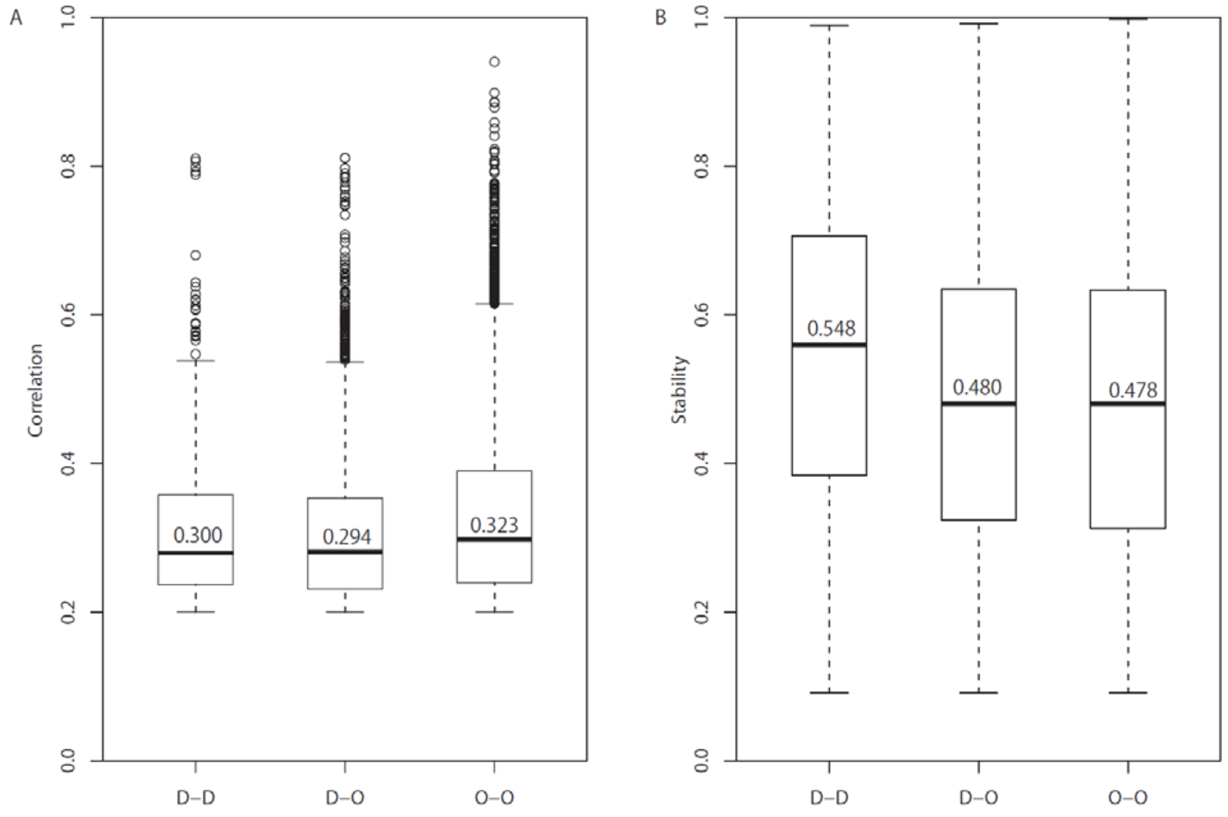

Figure S5. Coexpression correlation and stability for interacting protein pairs with varying levels of intrinsic disorder with average values specified in the boxplots. D-D: both interacting proteins have >=30% intrinsic disorder; D-O: one of the interacting proteins has >= 30% intrinsic disorder; O-O: both interacting proteins have less than 30% intrinsic disorder. The difference in the average correlation coefficient between D-D and O-O ($p = 0.0001$), and D-O and O-O ($p = 1.44e^{-13}$) is statistically significant. The difference in the average stability is significant between D-D and O-O ($p = 4.67e^{-15}$) and, D-D and D-O ($p = 1.44e^{-13}$). Statistical significance calculated by the Wilcoxon rank sum test.

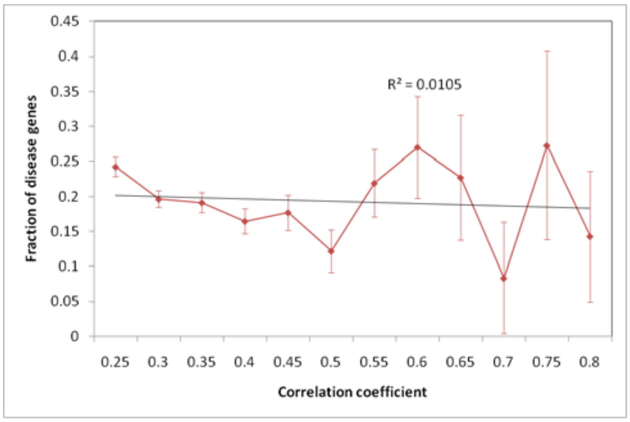

a)

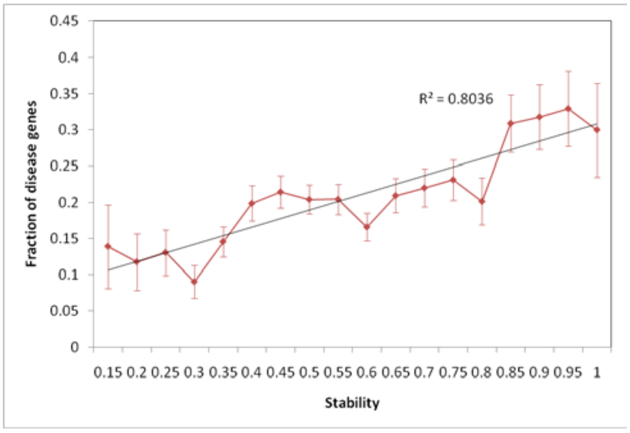

b)

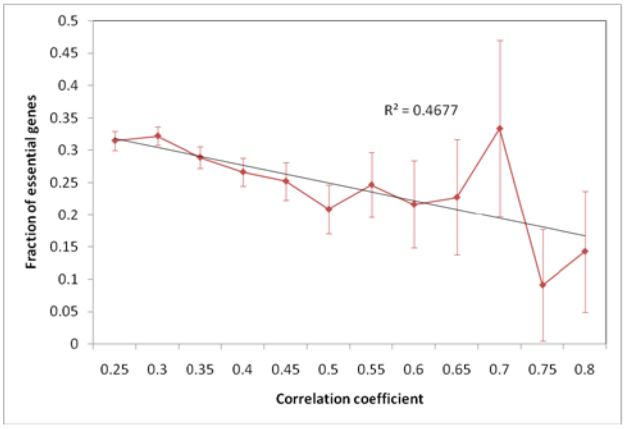

c)

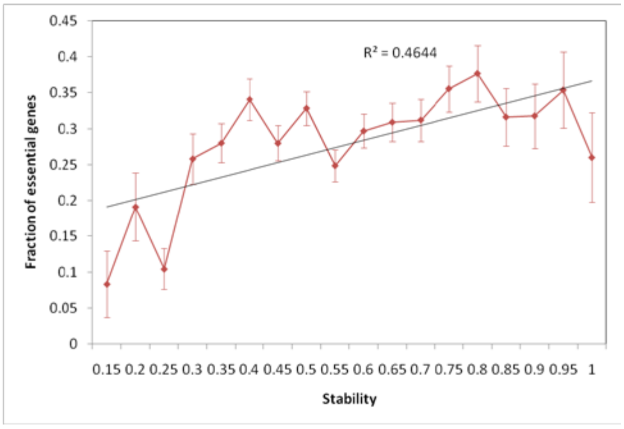

d)

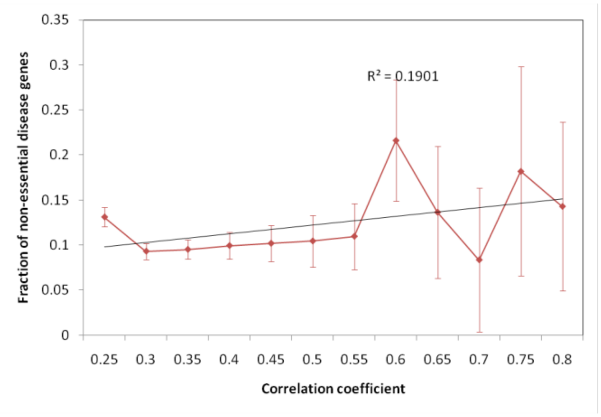

e)

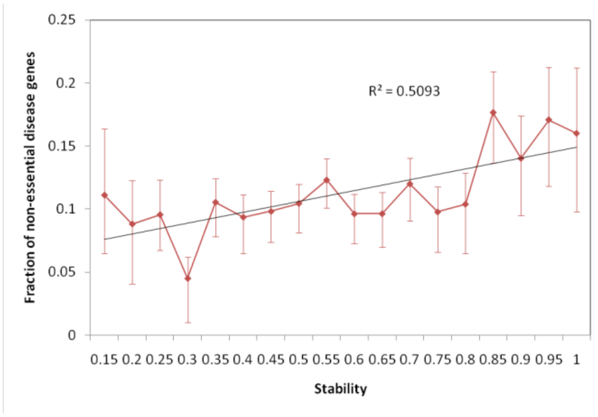

f)

Figure S6. Fraction of disease genes with the indicated average co-expression a) correlation and b)stability. Fraction of essential genes with the indicated average co-expression c) correlation and d) stability. Fraction of non-essential disease genes with the indicated average co-expression e) correlation and f) stability.

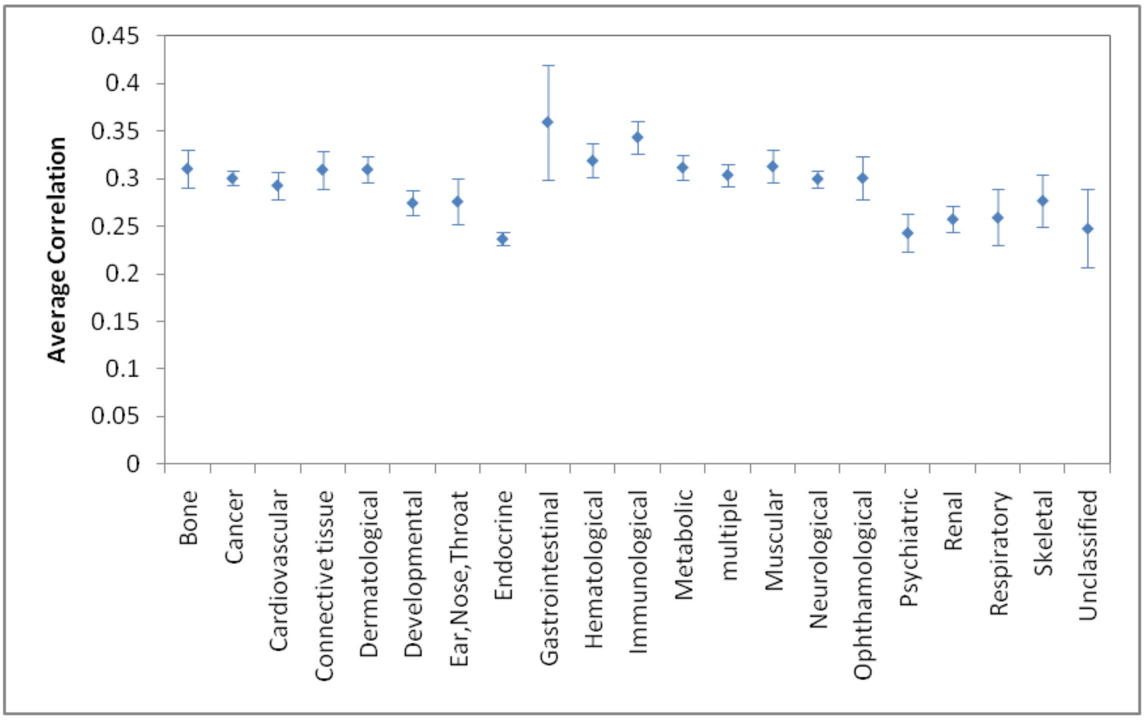

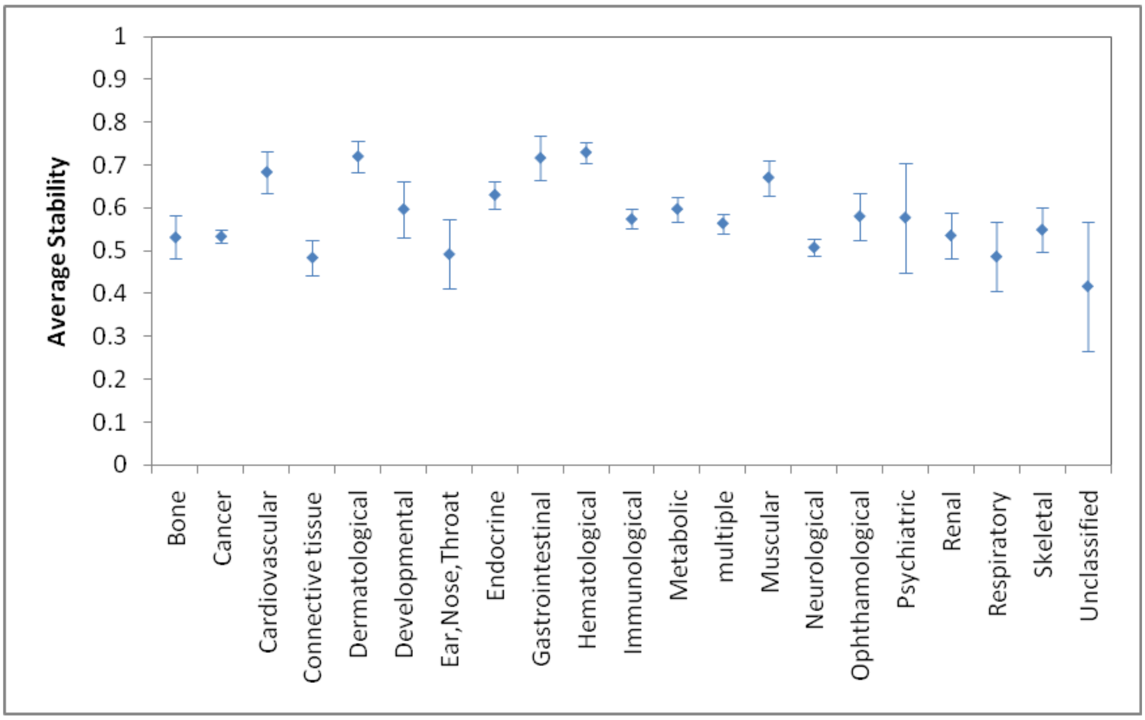

Figure S7. Average co-expression correlation and stability for genes in different disease classes. Error bars indicate standard error.

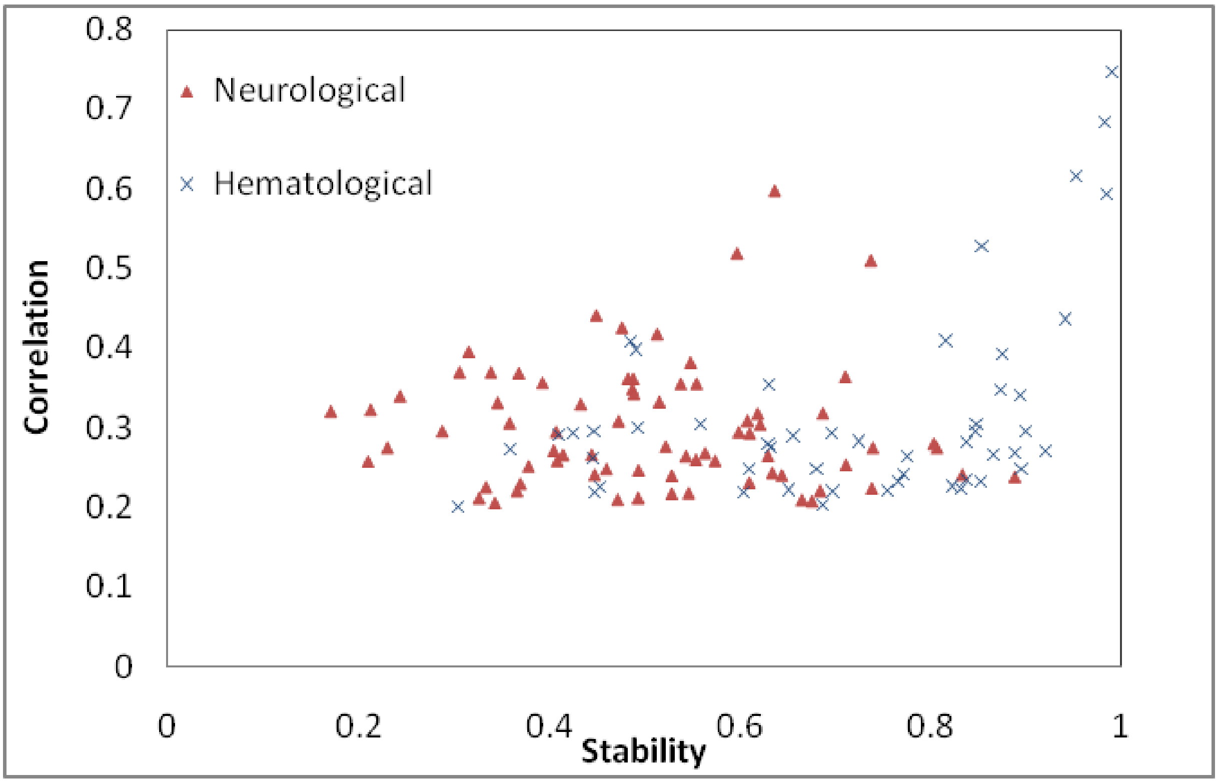
Neurological
Hematological
Correlation
Stability
0.8
0.7
0.6
0.5
0.4
0.3
0.2
0.1
0
0
0.2
0.4
0.6
0.8
1

a)

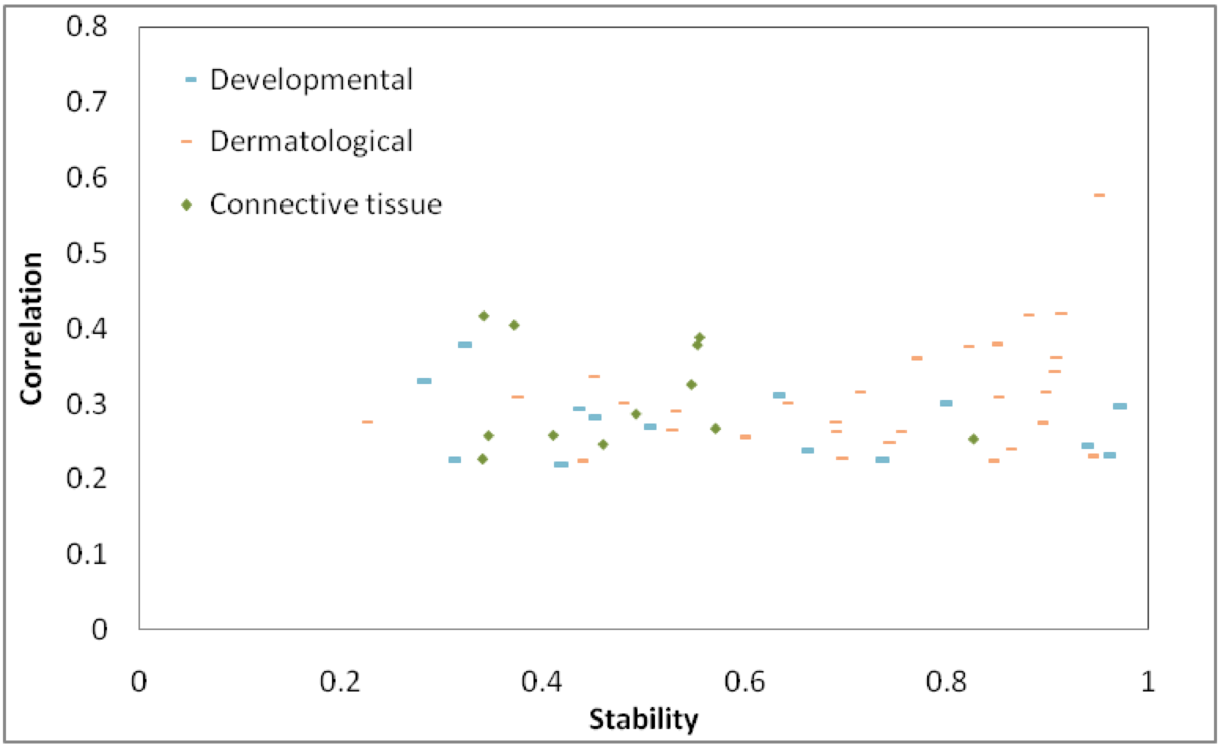
Developmental
Dermatological
Connective tissue
Correlation
Stability
0.8
0.7
0.6
0.5
0.4
0.3
0.2
0.1
0
0
0.2
0.4
0.6
0.8
1

b)

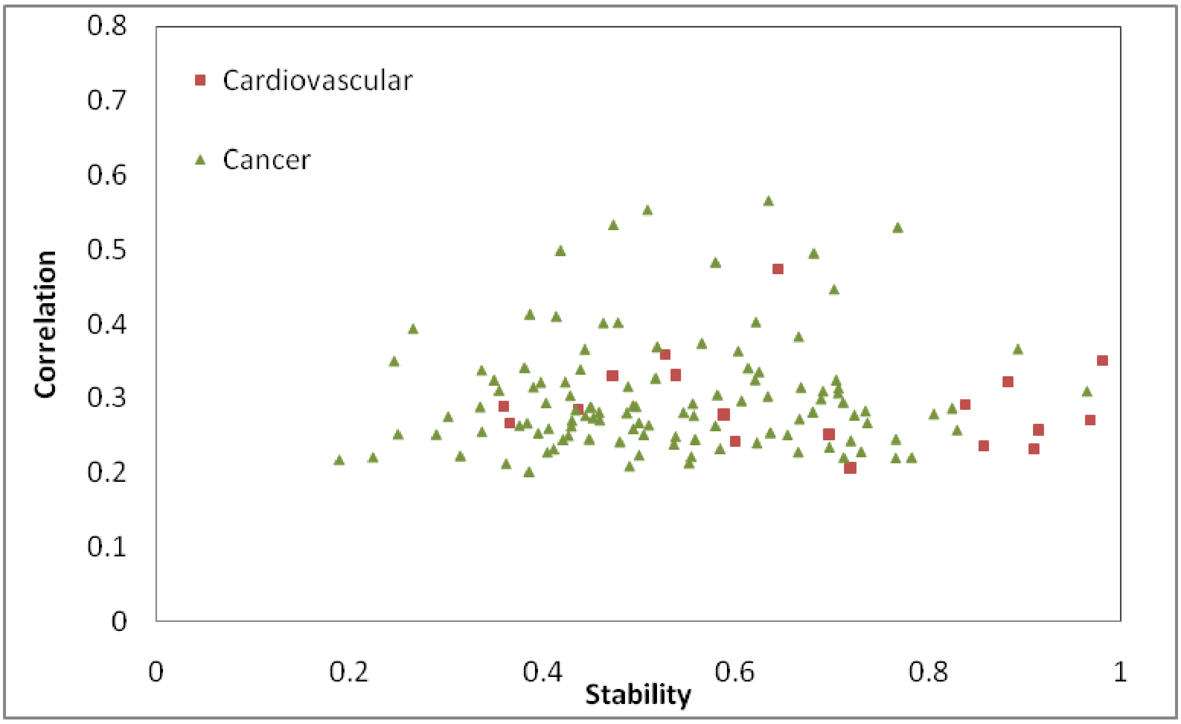

c)

Figure S8 Differences in patterns of average coexpression correlation and stability for genes implicated in a)neurological and hematological diseases, b)developmental, dermatological and connective tissue related diseases, and c) cardiovascular diseases and cancer.

Supplementary Tables

Interaction Category	Correlation	Stability	Number of interactions	Number of proteins	Number of unique hubs
1	> 0.5	> 0.5	564	530	49
2	<= 0.5	> 0.5	3026	2595	264
3	< =0.5	< =0.5	3416	2198	315
4	> 0.5	< =0.5	92	121	4

Table S1. Number of interactions, proteins and unique hubs in each category based on the coexpression correlation and stability of each pair of interacting proteins. Hubs in each category are defined as proteins that have at least 5 interactions that satisfy the conditions of coexpression correlation and stability with other proteins.

Table S2. Top GO Biological Process terms significantly enriched in hubs in different categories ($p < 0.01$)

**Category 1 hubs**		**Category 2 hubs**		**Category 3 hubs**	
GO:0031145	anaphase-promoting complex-dependent proteasomal ubiquitin-dependent protein catabolic process	GO:0044419	interspecies interaction between organisms	GO:0000398	nuclear mRNA splicing, via spliceosome
GO:0051437	positive regulation of ubiquitin-protein ligase activity during mitotic cell cycle	GO:0007265	Ras protein signal transduction	GO:0006367	transcription initiation from RNA polymerase II promoter
GO:0051436	negative regulation of ubiquitin-protein ligase activity during mitotic cell cycle	GO:0043123	positive regulation of I-kappaB kinase/NF-kappaB cascade	GO:0006368	RNA elongation from RNA polymerase II promoter
GO:0006270	DNA replication initiation	GO:0007242	intracellular signaling cascade	GO:0000387	spliceosomal snRNP biogenesis
GO:0007067	mitosis	GO:0006468	protein amino acid phosphorylation	GO:0006270	DNA replication initiation
GO:0000076	DNA replication checkpoint	GO:0000398	nuclear mRNA splicing, via spliceosome	GO:0051028	mRNA transport
GO:0007051	spindle organization	GO:0006916	anti-apoptosis	GO:0006397	mRNA processing
GO:0051301	cell division	GO:0010033	response to organic substance	GO:0006260	DNA replication
GO:0007049	cell cycle	GO:0048167	regulation of synaptic plasticity	GO:0006511	ubiquitin-dependent protein catabolic process

Table S3. Top GO Molecular Function terms significantly enriched in hubs in different categories (p < 0.01)

**Category 1 hubs**		**Category 2 hubs**		**Category 3 hubs**	
GO:0004298	threonine-type endopeptidase activity	GO:0005524	ATP binding	GO:0003723	RNA binding
GO:0017111	nucleoside-triphosphatase activity	GO:0004715	non-membrane spanning protein tyrosine kinase activity	GO:0005515	protein binding
GO:0005515	protein binding	GO:0008270	zinc ion binding	GO:0005524	ATP binding
GO:0005524	ATP binding	GO:0030235	nitric-oxide synthase regulator activity	GO:0003899	DNA-directed RNA polymerase activity
GO:0043142	single-stranded DNA-dependent ATPase activity	GO:0005057	receptor signaling protein activity	GO:0003688	DNA replication origin binding
GO:0000150	recombinase activity	GO:0019904	protein domain specific binding	GO:0000175	3'-5'-exoribonuclease activity
GO:0008121	ubiquinol-cytochrome-c reductase activity	GO:0005515	protein binding	GO:0016251	general RNA polymerase II transcription factor activity
GO:0050699	WW domain binding	GO:0016563	transcription activator activity	GO:0042802	identical protein binding
GO:0003677	DNA binding	GO:0003682	chromatin binding	GO:0047485	protein N-terminus binding

Table S4. Top GO Cellular Component terms significantly enriched in hubs in different categories ($p < 0.01$)

**Category 1 hubs**		**Category 2 hubs**		**Category 3 hubs**	
GO:0005839	proteasome core complex	GO:0005829	cytosol	GO:0005654	nucleoplasm
GO:0005654	nucleoplasm	GO:0005925	focal adhesion	GO:0005681	spliceosome
GO:0005829	cytosol	GO:0042470	melanosome	GO:0005829	cytosol
GO:0005634	nucleus	GO:0005886	plasma membrane	GO:0005643	nuclear pore
GO:0000775	chromosome, centromeric region	GO:0005884	actin filament	GO:0030532	small nuclear ribonucleoprotein complex
GO:0000794	condensed nuclear chromosome	GO:0005913	cell-cell adherens junction	GO:0030529	ribonucleoprotein complex
GO:0030532	small nuclear ribonucleoprotein complex	GO:0005667	transcription factor complex	GO:0005634	nucleus
GO:0042555	MCM complex	GO:0042101	T cell receptor complex	GO:0005669	transcription factor TFIID complex
GO:0051233	spindle midzone	GO:0005856	cytoskeleton	GO:0000178	exosome (RNase complex)

Interaction type	Count
D-D	685
D-O	2744
O-O	4753
Total	8182

Table S5. Number of interactions for each interaction type. D-D: both interacting proteins have >=30% intrinsic disorder; D-O: one of the interacting proteins has >= 30% intrinsic disorder; O-O: both interacting proteins have less than 30% intrinsic disorder.

Gene type	Essential	Non-essential	Total
Disease	350	398	748
Non-disease	746	2221	2967
Total	1096	2619	3715

Table S6. Number of disease, essential, non-disease and non-essential genes used in this study.
